# Redefining DNA cleavage in type I CRISPR systems with the HNH domain

**DOI:** 10.1002/mco2.70060

**Published:** 2025-02-13

**Authors:** Senfeng Zhang, Yao Liu, Chunyi Hu

**Affiliations:** ^1^ Department of Biological Sciences, Faculty of Science National University of Singapore Singapore Singapore; ^2^ Department of Biochemistry, Yong Loo Lin School of Medicine National University of Singapore Singapore Singapore; ^3^ Precision Medicine Translational Research Programme (TRP) National University of Singapore Singapore Singapore

1

Two recent cryo‐electron microscopy (cryo‐EM) studies have captured the HNH domain's ability to replace Cas3 within the Cascade complex (CRISPR‐associated complex for antiviral defense) in both type I‐E and type I‐F CRISPR‐Cas systems.^1^, ^2^ These findings reveal that Cas3 is not required and the HNH domain forms a ring‐like structure with Cascade, enabling precise DNA cleavage and providing a new avenue for understanding bacterial CRISPR‐Cas immune responses.

CRISPR‐Cas systems, which stand for Clustered Regularly Interspaced Short Palindromic Repeats and CRISPR‐associated genes, are adaptive immunity mechanisms in bacteria and archaea, designed responsible for protecting against viral invasion. The immune response typically involves three stages: adaptation, expression, and interference. Based on the Cas effectors used during CRISPR interference, these systems are categorized into two classes and seven types. Class 1, including types I, III, IV, and VII, comprises multi‐subunit Cas protein effector complexes, whereas Class 2, which includes types II, V, and VI, is composed of single‐subunit Cas protein effectors.^3^, ^4^

In canonical type I systems, multiple Cas proteins assemble into a crRNA‐containing Cascade complex, which stands for CRISPR‐associated complex for antiviral defense, with the exclusion of Cas3. When the target DNA is recognized and full R‐loop formation, Cas 3 is recruited as the catalytic module responsible for mediating target DNA cleavage.^5^ However, recent studies have identified two novel variant type I systems. In one variant, the type I‐E system, the C‐terminus of Cas5 is fused with an HNH domain, and in another variant, the type I‐F system, the HNH domain is inserted into the C‐terminus of Cas8 (Figure 1A). Despite lacking the Cas3 nuclease, the HNH‐Cascade can still cleave target DNA. This is especially intriguing, as in type II CRISPR‐Cas systems, the HNH nuclease domain is responsible for cleaving the DNA target strand in Cas9. Consequently, understanding the processes behind PAM recognition, crRNA guidance, and precise DNA cleavage in HNH‐mediated immunity within type I‐E and type I‐F systems has emerged as a critical area of research.

**FIGURE 1:**
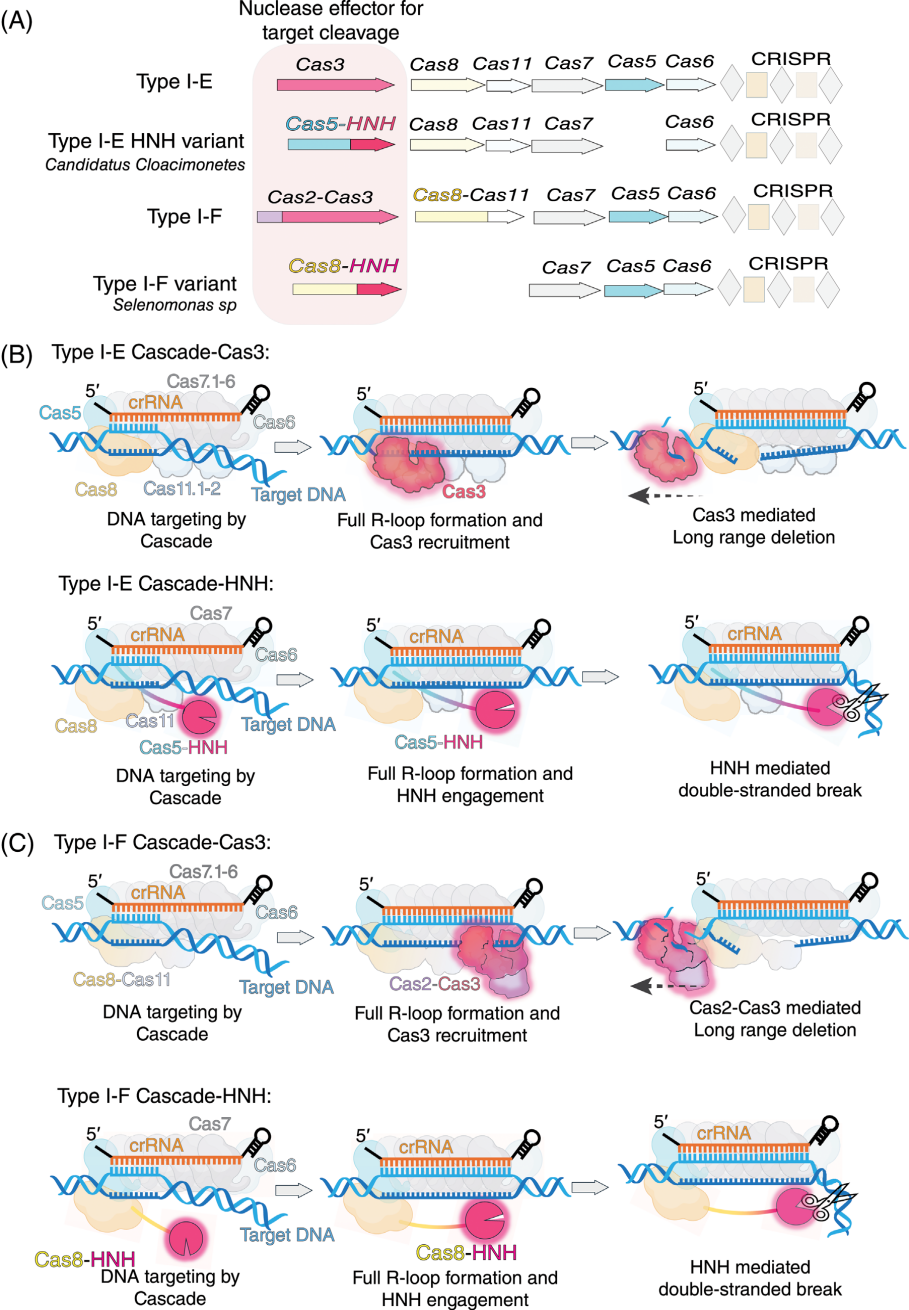
Structural and functional overview of type I‐E and type I‐F HNH‐cascade systems. (A) Schematic representation of the nuclease effectors for target DNA cleavage in type I CRISPR‐Cas systems. The diagram compares the canonical type I‐E and type I‐F systems, which utilize Cas3 for DNA cleavage, with their HNH variants. In the type I‐E HNH variant (*Candidatus Cloacimonetes*), the C‐terminus of Cas5 is fused with the HNH domain, while in the type I‐F HNH variant (*Selenomonas sp*), the HNH domain is inserted into the C‐terminus of Cas8. (B) Functional mechanism of DNA cleavage in type I‐E Cascade systems. The upper panel illustrates the traditional Cas3‐mediated DNA cleavage, where the full R‐loop formation recruits Cas3, leading to long‐range deletion. The lower panel depicts the HNH‐mediated mechanism in the type I‐E system, where the Cas5‐HNH domain facilitates precise double‐stranded DNA breaks, with the HNH domain playing a pivotal role in target cleavage after full R‐loop formation. (C) Functional mechanism of DNA cleavage in type I‐F Cascade systems. Similar to the type I‐E system, the upper panel shows the Cas3‐mediated cleavage in a traditional type I‐F system, with Cas2‐Cas3 recruited after R‐loop formation. The lower panel presents the HNH‐mediated DNA cleavage mechanism in the type I‐F HNH‐Cascade system, where Cas8‐HNH performs the cleavage, replacing the Cas3 function, and enabling precise double‐stranded breaks without long‐range deletions. [The image was created by BioRender].

The two papers published by Hirano et al. and Zhang et al. report cryo‐EM studies in the 3.0–3.48 Å range, capturing distinct states of the target‐free HNH‐Cascade and target‐bound HNH‐Cascade complexes in the type I‐F (*Selenomonas sp*) and type I‐E (*Candidatus Cloacimonetes*) systems, respectively. These studies reveal that both the type I‐F HNH‐Cascade complex and the type I‐E HNH‐Cascade complex adopt a ring‐like architecture that provides a clear view of how these systems achieve precise DNA cleavage (Figure 1B).

In the type I‐F HNH‐Cascade complex, the structure is organized into head, backbone, and tail regions, resembling the subunit arrangement of type I‐F Cascade complexes in *Pseudomonas aeruginosa* (PaCascade). The Cas8‐HNH domain replaces the Cas8‐HB domain (the C‐terminal helical bundle, equivalent to Cas11) and is positioned between the head and tail sections. Within the tail region, the Cas5‐Cas8 heterodimer binds the crRNA's 5′‐handle region along with the PAM‐containing DNA duplex, while the head of Cascade is mainly composed of Cas6 and the crRNA's 3′‐handle. Upon binding of the target DNA with crRNA, complete R‐loop formation shifts the Cascade head outward, enabling Cas8‐HNH to detach, rotate, and align for target DNA cleavage. Thus, Cas8 plays dual roles in PAM recognition and target cleavage in the type I‐F HNH‐Cascade system (Figure 1C). In contrast, the type I‐E HNH‐Cascade complex showcases the Cas5‐HNH domain integrating into the “inner belly” of the complex, sandwiched and stabilized by Cas6 and Cas11, with the catalytic cleft facing towards Cas11 and against Cas6. The binding of target DNA and the formation of the full R‐loop drive the movement of the crRNA 3′‐handle, Cas6, and the Cas5‐HNH domain together, widening the substrate channel formed by Cas5‐HNH and Cas11, where it plays a pivotal role in DNA cleavage (Figure 1B).

In brief, the HNH endonuclease domain substitutes for the Cas3 helicase‐nuclease, which is usually present in standard type I CRISPR‐Cas systems. This research underscores the crucial role of crRNA‐guided positioning in targeting DNA and reveals how the HNH domain enables precise DNA cleavage by facilitating target DNA exposure and cutting. Together with the Cascade scaffold, the HNH domain creates a positively charged groove that stabilizes the R‐loop and ensures accurate, sequential nicking of both DNA strands.

These findings open further exploration into the adaptability and evolution of CRISPR‐Cas systems. Future research could focus on uncovering the evolutionary processes that incorporated the HNH domain into these immune systems, shedding light on their dynamic adaptability. Additionally, studying other CRISPR‐Cas subtypes may reveal whether similar mechanisms exist and uncover alternative nuclease domains that contribute to DNA cleavage. The HNH‐Cascade system's precise DNA cleavage abilities hold promise for creating highly targeted genome‐editing tools, with potential applications in gene therapy and other therapeutics requiring precise DNA modifications. Moreover, assessing potential off‐target effects across different genomic contexts could enhance the safety and efficacy of these tools. In conclusion, the integration of the HNH domain within CRISPR‐Cas systems marks a breakthrough in understanding microbial immunity and unlocks new possibilities for biotechnological innovation.

## AUTHOR CONTRIBUTIONS

**Senfeng Zhang and Yao Liu**: wrote the manuscript. **Chunyi Hu**: drew the figure and approved the final version of the manuscript. All authors have read and approved the final manuscript.

## CONFLICT OF INTEREST STATEMENT

The authors declare no conflict of interest.

## ETHICS STATEMENT

Not applicable.

## Data Availability

Not applicable.
